# Supplementary figures and images for: Extending the audiogram with loudness growth: The complementarity of electric and acoustic hearing in bimodal patients
Source: PLoS One. 2023 Apr 20;18(4):e0277161. doi: 10.1371/journal.pone.0277161 (PMC10118154; doi:10.1371/journal.pone.0277161)

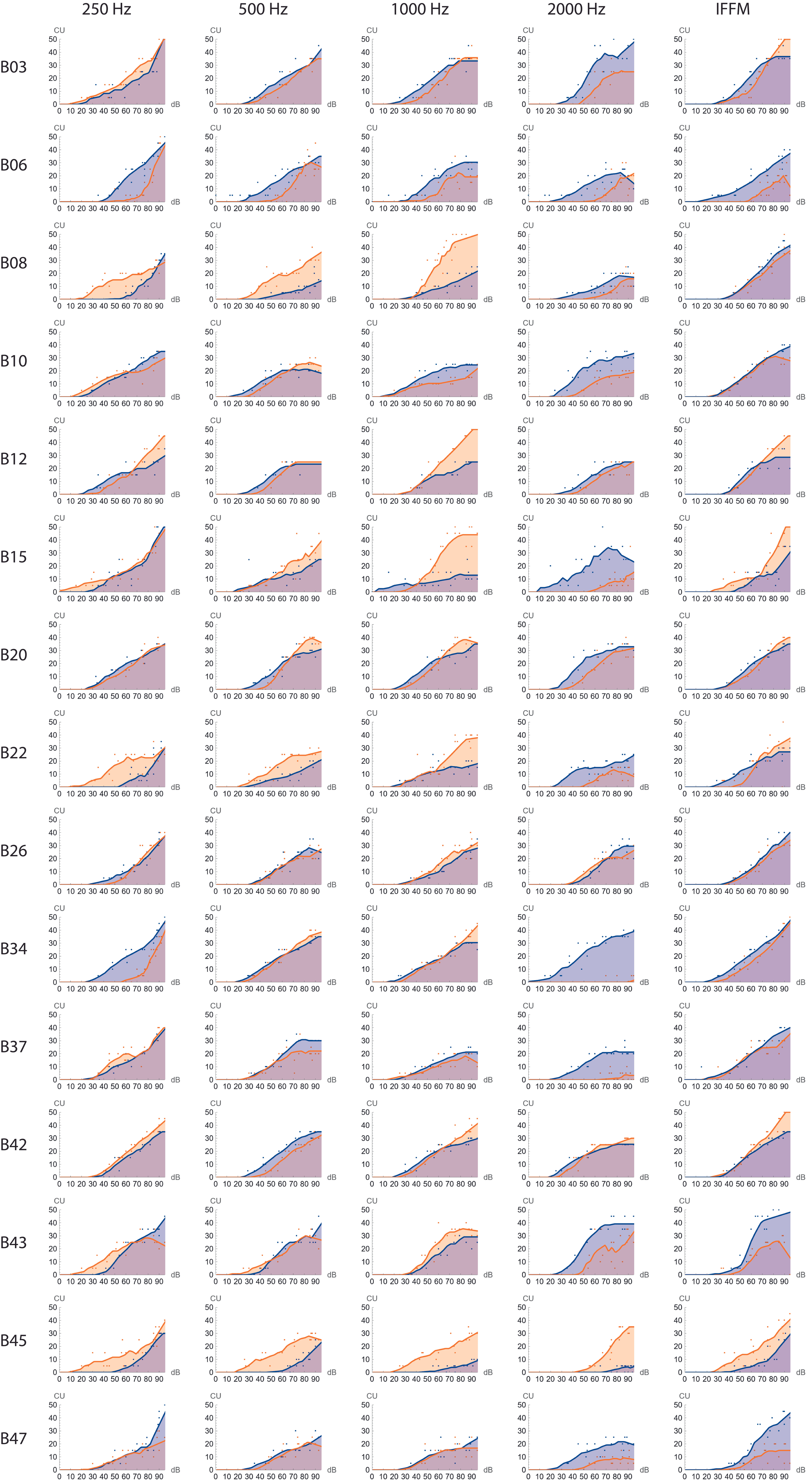

Supplement: S1 Fig — CI is shown in blue, HA in orange. Original ACALOS data are represented as dots. (TIF) [file pone.0277161.s001.tif]

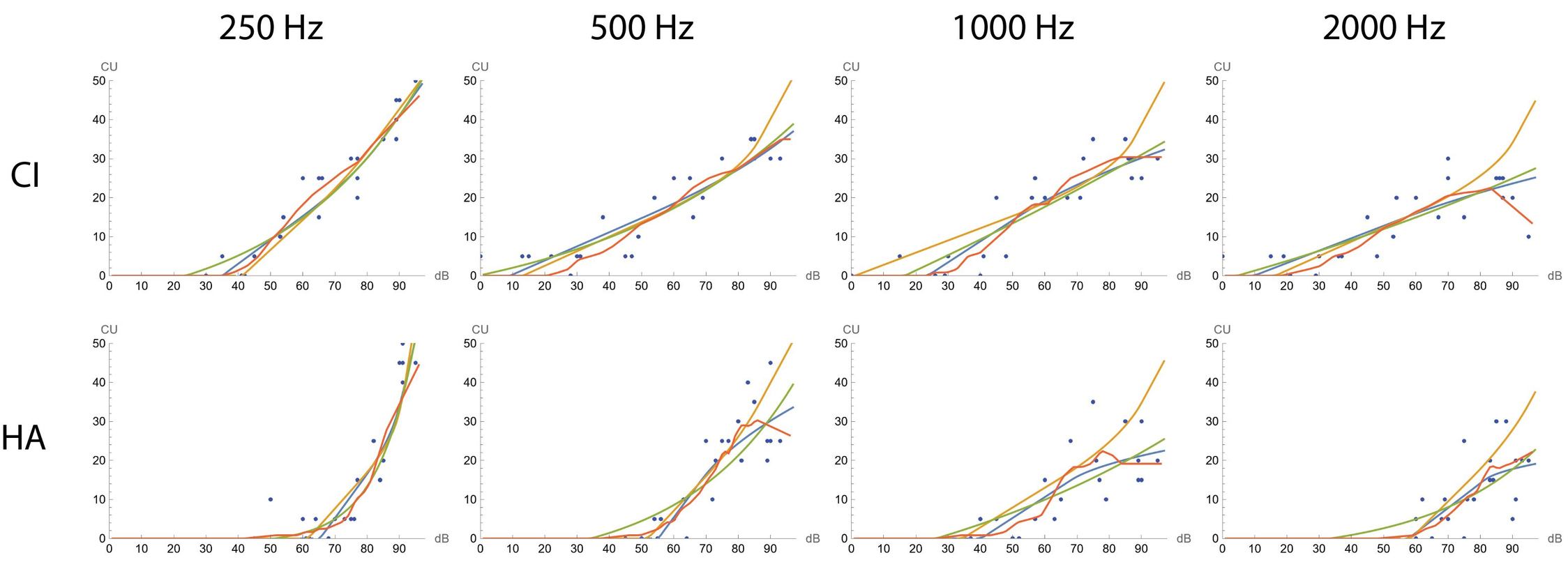

Supplement: S2 Fig — Fits are presented for aided measurements with CI and HA for one example patient (B06). (TIF) [file pone.0277161.s002.tif]
