# Supplementary material for: Extending the audiogram with loudness growth: The complementarity of electric and acoustic hearing in bimodal patients
Source: PLoS One. 2023 Apr 20;18(4):e0277161. doi: 10.1371/journal.pone.0277161 (PMC10118154; doi:10.1371/journal.pone.0277161)
Supplement: S1 Table — IQR = Interquartile Range. (DOCX) [file pone.0277161.s003.docx]

| ACALOS_NB_ | CI | HA | Overlap | CI+ | HA+ | Total CI+HA |
| --- | --- | --- | --- | --- | --- | --- |
| B03 | 98 | 80 | 78 | 20 | 2 | 100 |
| B06 | 100 | 55 | 55 | 45 | 0 | 100 |
| B08 | 39 | 98 | 38 | 2 | 61 | 100 |
| B10 | 96 | 72 | 69 | 28 | 4 | 100 |
| B12 | 78 | 92 | 70 | 8 | 22 | 100 |
| B15 | 66 | 88 | 54 | 12 | 34 | 100 |
| B20 | 92 | 87 | 79 | 13 | 8 | 100 |
| B22 | 60 | 94 | 54 | 6 | 40 | 100 |
| B26 | 91 | 94 | 85 | 6 | 9 | 100 |
| B34 | 95 | 72 | 67 | 28 | 5 | 100 |
| B37 | 98 | 73 | 71 | 27 | 2 | 100 |
| B42 | 93 | 87 | 80 | 13 | 7 | 100 |
| B43 | 87 | 88 | 76 | 12 | 13 | 100 |
| B45 | 26 | 100 | 26 | 0 | 74 | 100 |
| B47 | 99 | 77 | 77 | 23 | 1 | 100 |
| Median | 92 | 87 | 70 | 13 | 8 | 100 |
| IQR | 25 | 18 | 23 | 18 | 25 | 0 |

| ACALOS_BB_ | CI | HA | Overlap | CI+ | HA+ | Total CI+HA |
| --- | --- | --- | --- | --- | --- | --- |
| B03 | 90 | 85 | 75 | 15 | 10 | 100 |
| B06 | 100 | 33 | 33 | 67 | 0 | 100 |
| B08 | 100 | 87 | 87 | 13 | 0 | 100 |
| B10 | 100 | 89 | 89 | 11 | 0 | 100 |
| B12 | 83 | 95 | 78 | 5 | 17 | 100 |
| B15 | 53 | 100 | 53 | 0 | 47 | 100 |
| B20 | 94 | 91 | 86 | 9 | 6 | 100 |
| B22 | 87 | 83 | 70 | 17 | 13 | 100 |
| B26 | 100 | 86 | 86 | 14 | 0 | 100 |
| B34 | 100 | 80 | 80 | 20 | 0 | 100 |
| B37 | 99 | 86 | 85 | 14 | 1 | 100 |
| B42 | 79 | 100 | 79 | 0 | 21 | 100 |
| B43 | 100 | 47 | 47 | 53 | 0 | 100 |
| B45 | 45 | 100 | 45 | 0 | 55 | 100 |
| B47 | 100 | 41 | 41 | 59 | 0 | 100 |
| Median | 99 | 86 | 78 | 14 | 1 | 100 |
| IQR | 15 | 11,5 | 35,5 | 11,5 | 15 | 0 |
