# Supplementary material for: Extending the audiogram with loudness growth: The complementarity of electric and acoustic hearing in bimodal patients
Source: PLoS One. 2023 Apr 20;18(4):e0277161. doi: 10.1371/journal.pone.0277161 (PMC10118154; doi:10.1371/journal.pone.0277161)
Supplement: S2 Table — IQR = Interquartile Range. (DOCX) [file pone.0277161.s004.docx]

|  | 250 Hz | | | | | | | | | | | | 500 Hz | | | | | |
| --- | --- | --- | --- | --- | --- | --- | --- | --- | --- | --- | --- | --- | --- | --- | --- | --- | --- | --- |
|  | CI | | | HA | | Overlap | | CI+ | | HA+ | Total CI+HA | | CI | HA | Overlap | CI+ | HA+ | Total CI+HA |
| B03 | 81 | | | 100 | | 81 | | 0 | | 19 | 100 | | 100 | 83 | 83 | 17 | 0 | 100 |
| B06 | 100 | | | 46 | | 46 | | 54 | | 0 | 100 | | 100 | 61 | 60 | 39 | 0 | 100 |
| B08 | 47 | | | 98 | | 44 | | 2 | | 53 | 100 | | 27 | 100 | 27 | 0 | 73 | 100 |
| B10 | 91 | | | 88 | | 79 | | 12 | | 9 | 100 | | 90 | 87 | 77 | 13 | 10 | 100 |
| B12 | 80 | | | 88 | | 69 | | 12 | | 20 | 100 | | 96 | 87 | 83 | 13 | 4 | 100 |
| B15 | 85 | | | 98 | | 83 | | 2 | | 15 | 100 | | 72 | 97 | 69 | 3 | 28 | 100 |
| B20 | 98 | | | 91 | | 89 | | 9 | | 2 | 100 | | 87 | 88 | 75 | 12 | 13 | 100 |
| B22 | 40 | | | 100 | | 40 | | 0 | | 60 | 100 | | 51 | 100 | 51 | 0 | 49 | 100 |
| B26 | 95 | | | 90 | | 84 | | 10 | | 5 | 100 | | 99 | 90 | 89 | 10 | 1 | 100 |
| B34 | 100 | | | 39 | | 39 | | 61 | | 0 | 100 | | 93 | 95 | 88 | 5 | 7 | 100 |
| B37 | 86 | | | 100 | | 86 | | 0 | | 14 | 100 | | 100 | 78 | 78 | 22 | 0 | 100 |
| B42 | 81 | | | 100 | | 81 | | 0 | | 19 | 100 | | 100 | 71 | 71 | 29 | 0 | 100 |
| B43 | 81 | | | 88 | | 69 | | 12 | | 19 | 100 | | 93 | 90 | 83 | 10 | 7 | 100 |
| B45 | 50 | | | 100 | | 50 | | 0 | | 50 | 100 | | 31 | 100 | 31 | 0 | 69 | 100 |
| B47 | 99 | | | 78 | | 76 | | 22 | | 1 | 100 | | 99 | 85 | 84 | 15 | 1 | 100 |
| Median | 85 | | | 91 | | 76 | | 9 | | 15 | 100 | | 93 | 88 | 77 | 12 | 7 | 100 |
| IQR | 16 | | | 12 | | 34 | | 12 | | 16 | 0 | | 20 | 12 | 19 | 12 | 20 | 0 |
|  | | 1000 Hz | | | | | | | | | | 2000 Hz | | | | | | |
| B03 | | 98 | 81 | | 79 | | 19 | | 2 | | 100 | 100 | | 49 | 49 | 51 | 0 | 100 |
| B06 | | 100 | 56 | | 56 | | 44 | | 0 | | 100 | 96 | | 43 | 40 | 57 | 4 | 100 |
| B08 | | 36 | 100 | | 35 | | 0 | | 64 | | 100 | 100 | | 50 | 50 | 50 | 0 | 100 |
| B10 | | 100 | 62 | | 62 | | 38 | | 0 | | 100 | 100 | | 44 | 44 | 56 | 0 | 100 |
| B12 | | 58 | 100 | | 58 | | 0 | | 42 | | 100 | 100 | | 79 | 79 | 21 | 0 | 100 |
| B15 | | 39 | 93 | | 32 | | 7 | | 61 | | 100 | 100 | | 18 | 18 | 82 | 0 | 100 |
| B20 | | 90 | 91 | | 80 | | 9 | | 10 | | 100 | 100 | | 71 | 71 | 29 | 0 | 100 |
| B22 | | 62 | 99 | | 61 | | 1 | | 38 | | 100 | 100 | | 38 | 38 | 62 | 0 | 100 |
| B26 | | 82 | 99 | | 81 | | 1 | | 18 | | 100 | 92 | | 88 | 80 | 12 | 8 | 100 |
| B34 | | 91 | 95 | | 86 | | 5 | | 9 | | 100 | 100 | | 0 | 0 | 100 | 0 | 100 |
| B37 | | 98 | 79 | | 77 | | 21 | | 2 | | 100 | 100 | | 6 | 6 | 94 | 0 | 100 |
| B42 | | 88 | 94 | | 82 | | 6 | | 12 | | 100 | 93 | | 93 | 86 | 7 | 7 | 100 |
| B43 | | 74 | 100 | | 74 | | 0 | | 26 | | 100 | 100 | | 49 | 49 | 51 | 0 | 100 |
| B45 | | 16 | 100 | | 16 | | 0 | | 84 | | 100 | 9 | | 100 | 9 | 0 | 91 | 100 |
| B47 | | 100 | 89 | | 89 | | 11 | | 0 | | 100 | 100 | | 33 | 33 | 67 | 0 | 100 |
| Median | | 88 | 94 | | 74 | | 6 | | 12 | | 100 | 100 | | 49 | 44 | 51 | 0 | 100 |
| IQR | | 38 | 15 | | 24 | | 15 | | 38 | | 0 | 2 | | 39 | 35 | 39 | 2 | 0 |
