# Supplementary material for: Extending the audiogram with loudness growth: The complementarity of electric and acoustic hearing in bimodal patients
Source: PLoS One. 2023 Apr 20;18(4):e0277161. doi: 10.1371/journal.pone.0277161 (PMC10118154; doi:10.1371/journal.pone.0277161)
Supplement: S3 Table — IQR = Interquartile Range. (DOCX) [file pone.0277161.s005.docx]

|  | Speech in quiet | | | |  | Speech in noise | | | |
| --- | --- | --- | --- | --- | --- | --- | --- | --- | --- |
|  | CI | HA | CIHA | Bimodal benefit | CI | HA | CIHA | Bimodal benefit | |
|  | % correct | % correct | % correct | Normalized benefit | iSRT (dB SNR) | iSRT (dB SNR) | iSRT (dB SNR) | Summation (iSRT, dB SNR) | Head shadow (iSRT, dB SNR) |
| B03 | 0 | 52 | 55 | 55 | - | NM | -3,10 | - | - |
| B06 | 72 | 27 | 69 | -4 | -9,3 | NM | -8,10 | 1,2 | 1,35 |
| B08 | 57 | 51 | 72 | 35 | -12,3 | NM | -2,90 | 9,4 | 9 |
| B10 | 72 | 54 | 81 | 32 | -3,4 | NM | 2,50 | 5,9 | 2,45 |
| B12 | 45 | 39 | 60 | 27 | - | NM | -6,90 | - | - |
| B15 | 67 | 61 | 79 | 36 | -8,5 | NM | -0,50 | 8 | 9,65 |
| B20 | 85 | 52 | 79 | -7 | 3,2 | NM | 4,80 | 1,6 | 1,6 |
| B22 | 82 | 42 | 91 | 50 | -5,4 | NM | -0,10 | 5,3 | 12,5 |
| B26 | 57 | 75 | 84 | 63 | -2,7 | NM | 3,40 | 6,1 | 9,35 |
| B34 | 64 | 48 | 79 | 42 | -2,4 | NM | -0,80 | 1,6 | 2,95 |
| B37 | 66 | 30 | 69 | 9 | -0,1 | NM | 1,50 | 1,6 | 3,85 |
| B42 | 96 | 78 | 100 | 100 | 2,3 | NM | 5,70 | 3,4 | 6,75 |
| B43 | 39 | 49 | 58 | 31 | - | NM | -14,60 | - | - |
| B45 | 24 | 51 | 63 | 51 | - | NM | -2,70 | - | - |
| B47 | 73 | 36 | 79 | 22 | 5,8 | NM | 3,20 | 2,6 | 5,4 |
| Median | 66 | 51 | 79 | 35 | -2,7 | NM | -0,5 | 3,4 | 5,4 |
| IQR | 19 | 12 | 13 | 25 | 8,1 | NM | 5,9 | 4,4 | 6,5 |
