# Supplementary material for: Extending the audiogram with loudness growth: The complementarity of electric and acoustic hearing in bimodal patients
Source: PLoS One. 2023 Apr 20;18(4):e0277161. doi: 10.1371/journal.pone.0277161 (PMC10118154; doi:10.1371/journal.pone.0277161)
Supplement: S4 Table — Dynamic range was averaged across frequencies and is shown for CI, HA and as a difference score between the two devices. (DOCX) [file pone.0277161.s006.docx]

|  | CI | HA | Difference (HA - CI) |
| --- | --- | --- | --- |
| B03 | 58,28 | 51,90 | -6,38 |
| B06 | 53,16 | 25,61 | -27,55 |
| B08 | 37,51 | 52,19 | 14,68 |
| B10 | 61,70 | 59,76 | -1,94 |
| B12 | 51,95 | 46,45 | -5,5 |
| B15 | 58,11 | 52,52 | -5,59 |
| B20 | 62,00 | 49,34 | -12,66 |
| B22 | 53,04 | 53,67 | 0,63 |
| B26 | 45,39 | 49,79 | 4,4 |
| B34 | 65,07 | 37,13 | -27,94 |
| B37 | 47,67 | 37,76 | -9,91 |
| B42 | 58,68 | 56,14 | -2,54 |
| B43 | 49,78 | 48,07 | -1,71 |
| B45 | 19,14 | 58,04 | 38,9 |
| B47 | 54,92 | 40,97 | -13,95 |
| Median | 53,16 | 49,79 | 5,50 |
| IQR | 9,76 | 9,34 | 10,75 |
