# Supplementary material for: Extending the audiogram with loudness growth: The complementarity of electric and acoustic hearing in bimodal patients
Source: PLoS One. 2023 Apr 20;18(4):e0277161. doi: 10.1371/journal.pone.0277161 (PMC10118154; doi:10.1371/journal.pone.0277161)
Supplement: S5 Table — (DOCX) [file pone.0277161.s007.docx]

|  |  |  | Loudness growth (BB) | | | | | |  |
| --- | --- | --- | --- | --- | --- | --- | --- | --- | --- |
|  |  |  | CI | HA | Overlap | CI+ | HA+ | Total CI+HA | |
| Loudness growth (NB) | 250 Hz | Rho | 0,46 | 0,49 | 0,25 | 0,56 | 0,56 | 0,67 | |
|  |  | P | 0,08 | 0,07 | 0,36 | 0,03* | 0,03* | 0,01* | |
|  | 500 Hz | Rho | 0,57 | 0,23 | 0,61 | 0,18 | 0,22 | 0,49 | |
|  |  | P | 0,03* | 0,41 | 0,02* | 0,53 | 0,43 | 0,07 | |
|  | 1000 Hz | Rho | 0,62 | 0,29 | 0,53 | 0,18 | 0,51 | 0,09 | |
|  |  | P | 0,01* | 0,30 | 0,04* | 0,53 | 0,05 | 0,74 | |
|  | 2000 Hz­ | Rho | 0,64 | 0,15 | 0,09 | 0,48 | 0,23 | 0,63 | |
|  |  | P | 0,01* | 0,58 | 0,74 | 0,07 | 0,42 | 0,01* | |
