# Supplementary material for: Extending the audiogram with loudness growth: The complementarity of electric and acoustic hearing in bimodal patients
Source: PLoS One. 2023 Apr 20;18(4):e0277161. doi: 10.1371/journal.pone.0277161 (PMC10118154; doi:10.1371/journal.pone.0277161)
Supplement: Attachment — Submitted filename: Response to reviewers.docx [file pone.0277161.s009.docx]

**Reviewer #1**

• Please ensure that your manuscript meets PLOS ONE's style requirements, including those for file naming.

**The new manuscript has been revised according to the PLOS ONE style templates. Specifically, we corrected the file names, removed the section numbers out of the headers and improved table formatting. Officially, Table 2 might not meet journal requirements due to figures inserted within the table. We have contacted Plos One productions on this matter, but have not yet received a response. If the table turns out to violate journal requirements, we would be happy to add the figures as supporting information or transform the table into a figure for the final version of the manuscript.**

• In your Data Availability statement, you have not specified where the minimal data set underlying the results described in your manuscript can be found. PLOS defines a study's minimal data set as the underlying data used to reach the conclusions drawn in the manuscript and any additional data required to replicate the reported study findings in their entirety. All PLOS journals require that the minimal data set be made fully available. For more information about our data policy, please see http://journals.plos.org/plosone/s/data-availability.

Upon re-submitting your revised manuscript, please upload your study’s minimal underlying data set as either Supporting Information files or to a stable, public repository and include the relevant URLs, DOIs, or accession numbers within your revised cover letter. For a list of acceptable repositories, please see http://journals.plos.org/plosone/s/data-availability#loc-recommended-repositories. Any potentially identifying patient information must be fully anonymized.

Important: If there are ethical or legal restrictions to sharing your data publicly, please explain these restrictions in detail. Please see our guidelines for more information on what we consider unacceptable restrictions to publicly sharing data: http://journals.plos.org/plosone/s/data-availability#loc-unacceptable-data-access-restrictions. Note that it is not acceptable for the authors to be the sole named individuals responsible for ensuring data access.

We will update your Data Availability statement to reflect the information you provide in your cover letter.

**In the revised submission of the manuscript, we have now added all data underlying the results as supporting information. As also referred to in the manuscript text, speech outcomes are now presented in S5 and loudness data in S8. With these datasets, it is possible to replicate our results.**

• Please include your full ethics statement in the ‘Methods’ section of your manuscript file. In your statement, please include the full name of the IRB or ethics committee who approved or waived your study, as well as whether or not you obtained informed written or verbal consent. If consent was waived for your study, please include this information in your statement as well.

**The following section was added under ‘Ethics’ to the methods section:**

**“This study has been approved by the ethics committee of the Maastricht University Medical Center (MUMC+) under registration number NL42011.068.13 and has been registered in the Dutch National Trial Register (NTR3932). Subjects provided written informed consent before participation and were compensated for their travelling costs.”**

• The paper is long. Ease of reading may be better if the aims are better defined. The authors have measured bimodal benefit using speech intelligibility at different SNRs, quality of speech, which is mentioned much later.

**In the revised manuscript, the introduction section has been rewritten. Now, the aims have been redefined and speech outcomes are introduced.**

**“This study had multiple aims. 1) To explore loudness growth differences between modalities (CI vs HA) in a group of bimodal subjects. 2) To relate loudness growth to speech recognition outcomes, such as speech intelligibility in quiet and noise, listening ease, and sound quality. These will be assessed both within the same listening condition (e.g. loudness growth with CI and speech recognition with CI) and bimodally (taking into account different properties of loudness across CI and HA, e.g. the extent to which HA was louder than CI and bimodal benefit). 3) Evaluate the loudness audiogram and analyze the additional benefit of measuring the full course of loudness growth compared to dynamic range (MCL-threshold). 4) Evaluate if measuring time of loudness scaling can be reduced by using broadband instead of narrowband stimuli.”**

• Clarity needs to be improved.- I have added some excerpts from the text to illustrate.
1.Aim in abstract: Clinically, the audiogram is the most commonly used measure when evaluating hearing loss and fitting hearing aids. As an extension, we present the loudness audiogram, which does not only show auditory thresholds but also visualizes the full course of loudness perception.

**This section has been revised to:**

**“Introduction: Clinically, recording hearing detection thresholds and representing them in an audiogram is the most common way of evaluating hearing loss and starting the fitting of hearing devices. As an extension, we present the loudness audiogram, which does not only show auditory thresholds but also visualizes the full course of loudness growth across frequencies. The additional benefit of this approach was evaluated in subjects who rely on both electric (cochlear implant) and acoustic (hearing aid) hearing.”**

• 2.Aim –lines 145-150 -The main goal of this study was to measure, visualize and compare loudness growth with both modalities (CI and HA) in a group of bimodal subjects and explore the relationship between bimodal loudness growth and bimodal speech performance. As a subgoal, to evaluate whether loudness growth provided distinctive information compared to threshold estimation alone, loudness was compared with audiometric thresholds and dynamic range. Also, to assess if measuring time for loudness scaling can be reduced, results of loudness growth measured with broadband and narrowband stimuli were compared.

**The section has been revised to:**

**“This study had multiple aims. 1) To explore loudness growth differences between modalities (CI vs HA) in a group of bimodal subjects. 2) To relate loudness growth to speech recognition outcomes, both within the same listening condition (e.g. loudness growth with CI and speech recognition with CI) and bimodally (taking into account different properties of loudness across CI and HA, e.g. the extent to which HA was louder than CI and bimodal benefit). 3) Evaluate the loudness audiogram and analyze the additional benefit of measuring the full course of loudness growth compared to dynamic range (MCL-threshold). 4) Evaluate if measuring time of loudness scaling can be reduced by using broadband instead of narrowband stimuli.”**

• There are some parts that are slightly redundant- For e.g .Line 235 mentions functions evaluated- but line 250 gives a better description and may be given in the beginning of the methods section. A flow chart would add to ease of reading.

**This section has been revised accordingly. Also, a table (Table 1) has been added to provide an overview of outcome measures.**

• A conclusion may be added to the abstract.

**In the revised abstract, we have merged discussion and conclusion to the following:**

**“****Conclusion: Results show that loudness growth is related to bimodal benefit for speech recognition in noise and to some aspects of speech quality. Subjects who had different input from the hearing aid compared to CI, generally gained more bimodal benefit compared to those patients whose hearing aid provided mainly equivalent input. This suggest that bimodal fitting to create equal loudness at all frequencies may not always be beneficial for speech recognition.”**

• Introduction needs to be more focused and shortened.
 **The introduction has been substantially shortened in the revised version of the manuscript. We believe the new introduction is more to-the-point and redundant information has been removed.**

• Methods: Loudness scaling was carried out with the device in the ear as mentioned in line 166. .“All measurements were performed in a sound attenuated booth with subjects using their own hearing devices at daily use settings with no manipulations allowed during testing.” The device was used in the everyday condition. How was bimodal fitting achieved? Were the devices matched for loudness? In hearing aids, the compression is set already, so it would have affected the loudness scaling. What was the prescription method used? Those settings need to be given. I notice that loudness for 250 Hz continues to be steep and may possibly be explained by those settings, and possibly the hearing thresholds of the participants, which show a great variability.

**In the revised version of the manuscript, we have explained hearing aid settings and fitting procedures in more detail. In general, it is good to clarify that subjects were part of a clinical population of bimodal users. Fitting of the hearing aids was already performed in a previous stage and according to clinical routine. In some cases, this was done in our own clinic, but more often subjects visited their local hearing aid professional for gain adjustments. As subjects used different brands of hearing devices (see Devocht et al. (reference number 17) for details), and normally the fitting rule of the hearing aid manufacturer was used, different fitting rules have been applied. Since we wanted to specifically evaluate the clinical performance of our subjects, we have not altered these settings and used daily use settings. We have tried to explain this in more detail in the corresponding methods section:**

**“CI fitting was performed according to clinical routine with real life adjustments based on behavioral M and T levels. All participants declared consistent use of a contralateral HA with different brands worn between subjects. During testing, they used their own hearing devices, at typical daily use settings and manipulations during the course of testing were not allowed. Hearing aids were fitted separately from CI, as part of clinical routine, either within the clinic or at a local hearing aid professional. No systematic bimodal fitting protocol was applied, as no generally accepted bimodal fitting methods exist. Both the CI speech processor and the HA were checked to ensure they were working correctly”.**

• Figure 1 and as seen in Devocht et al.( 2017). It may also expected, that the loudness with CI would never reach very loud because of the setting of the maximum level of stimulation.
In the light of the previous statement, the lines in 203-207 are confusing. “Both hearing aids and cochlear implants can alter the shape of the loudness curve due to individual settings such as compression rules and output limiting functionalities [45,46]. Therefore, in this study a newly developed loudness function was introduced. It aims for higher accuracy and less bias compared to current fitting functions when loudness scaling is performed in free field using hearing aids or cochlear implants. A detailed step-by-step description of the fit is shown in Table 1. The loudness function was programmed in Mathematica 12.3 (Wolfram Research, Champaign, USA)”

**We agree that the technical settings of both CI’s and hearing aids can affect the measured loudness curves. Maximum comfortable levels in the CI, and output limiting in the HA, can be expected to influence higher loudness levels as these will not be reached. We have noticed that the current loudness functions that are commonly used, are not suitable for aided measurements in free field (see supporting figure S2). Therefore, we have developed a new function that is more accurate in these conditions. In the paragraph starting at line 202 we have tried to clarify this. Here, we have now revised the phrasing to:**

**“Both hearing aids and cochlear implants can alter the shape of the loudness curve due to individual settings (i.e. maximum comfortable levels), compression rules and output limiting functionalities (Dillon, 2012; Theelen-van den Hoek et al., 2014).”**

• Results may be presented in terms of the aims. The order of presentation may be mentioned.

**Thanks to the suggestions of the reviewer, we believe the aims have now been better described in the introduction. We have also considered incorporating the aims as subheadings in the results section. However, we believe the current structure suits the manuscript somewhat better. In general, the order of the newly defined aims does correspond with the order of how the results are currently presented in the revised manuscript. Ofcourse, if the reviewers believe the manuscript would benefit from revising the order in the results section, we would be happy to incorporate this in the final version of the manuscript.**

• Table 1 is nicely designed with the function and figures put together. However the figures inserted in the table seem incomplete. -e.g.Table1B1p2.tif does not have any markings on the axis or abscissa and does not give a complete picture inspite of the explanation-“values of all cases where Y, loudness perception CU is zero”. Section D- Area under the surface calculation also needs a result.

**In the revised version of Table 2, figures have now been complemented with axes that show markings and explain categorical units. The result of the Area under the surface calculation has been noted under section D.**

• Speech recognition, speech understanding and “speech” have been used synonymously –speech recognition is more appropriate

**Throughout the revised manuscript, ‘speech recognition’ has now been used consistently.**

• Line 235-line 250 similar, better defined in 235ne 240 ‘ Threshold is misspelt

**This section has been revised to:**

**“Relationships between loudness and speech outcomes were studied for both performance within the same listening condition and bimodal benefit. Bimodal benefit was defined as the difference in speech outcome results between CIHA and CI alone. Only those outcomes (or subdomains) that showed significant bimodal benefit in Devocht et al. (Devocht et al., 2017) were included for analysis in this study. These included: CNC word recognition, sentence intelligibility in noise (inverted SRT (iSRT), in different spatial conditions), ease of listening (inverse of listening effort) at SRT +9 dB SNR, and sound quality for the ratings Full, Pleasant (inverse of Unpleasant) and Tinny.”.**

**The misspeling of ‘threshold’ has also been revised.**

• Line 285- Median ratio’s of loudness- does not need an apostrophe

**Has been revised accordingly.**

• Tables must match format of the journal. Tables 2, 3, 4 have a very long titles. Can some part be added as a footnote? Value of N number of subjects may be indicated in the table and reasons added in the footnote.

**In the revised manuscript, it has been ensured that tables now match the format of the journal. Long titles have been added as a footnote. The number of subjects is now mentioned in the table instead of in the legend, with commentary on differences in sample size between measurements now explained in the footnote.**

• In the tables, commas are used instead of a point to indicate probability or Rho values. If space permits, participant details may be given.

**In the revised manuscript, commas have now been replaced by points. Regarding the next comment, we would like to ask the reviewer which participant details are referred to. As referred to in the Subjects section in the methods, additional participant details can be found in Devocht et al. (reference number 17). Individual results in loudness and speech outcomes are now added as supporting information.**

**Reviewer #2**

• Standard deviation of age of the participants needs to be mentioned

**Standard deviations of age have now been added to line 149.**

• More information on the aided performance participants on the cochlear implant side

**Aided performance with both the cochlear implant and the hearing aid can be found in supporting table S5. Also, we have added the original loudness data as a datasheet in S8.**

• Information on details of programming of hearing aids/gain settings

**As mentioned as a response to a comment by reviewer 1, we have provided more details on fitting procedures and hearing aid settings in the revised version of the manuscript. Specifically, from line 152 onwards it now reads:**

**“CI fitting was performed according to clinical routine with real life adjustments based on behavioral M and T levels. All participants declared consistent use of a contralateral HA with different brands worn between subjects. During testing, they used their own hearing devices, at typical daily use settings and manipulations during the course of testing were not allowed. Hearing aids were fitted separately from CI, as part of clinical routine, either within the clinic or at a local hearing aid professional. No systematic bimodal fitting protocol was applied, as no generally accepted bimodal fitting methods exist. Both the CI speech processor and the HA were checked to ensure they were working correctly”.**
