# Supplementary material for: Extending the audiogram with loudness growth: The complementarity of electric and acoustic hearing in bimodal patients
Source: PLoS One. 2023 Apr 20;18(4):e0277161. doi: 10.1371/journal.pone.0277161 (PMC10118154; doi:10.1371/journal.pone.0277161)
Supplement: Attachment — Submitted filename: Reviews comments PONE-D-22-20984-R1 2023.docx [file pone.0277161.s010.docx]

**Review for:**

**Extending the audiogram with loudness growth: revealing complementarity 1 in bimodal aiding**

**Submitted to PLOS ONE**

**Manuscript Number: PONE-D-22-20984-R1**

**Comments to the associate editor**

Thank you for the opportunity to review this revised article. The motivation for the study is convincing and has good clinical implication. The paper has good writing. If revised with minor changes, such a paper will add value to the existing literature. With this intent, my review here attempts to highlight some of the minor issues and indicate the errors that are reflected in writing. I sincerely hope that the authors find this helpful in refining their manuscript.

**Summary**

The major objectives of this study were to obtain loudness audiogram, find the relationship between loudness growth and speech recognition in quiet, in noise and speech quality in CI alone, hearing aid alone, and CI+HA conditions, and to find out whether equal input from CI and HA leads to better speech recognition or not. The study included 15 bimodal users. Loudness growth was measured with the cochlear implant and 20 hearing aid separately using a loudness scaling procedure. The results showed that loudness growth was related to bimodal benefit for speech recognition in noise and some aspects of speech quality. Gain/mapping settings leading to best speech recognition are better than setting giving equal input from both devices.

Overall Impression (General Comments)

The strength of the manuscript is the motivation of the study and the flow in writing. The introduction section is adequate. The participants, materials, and procedures are explained well. However, the main drawback of the study is the small number of participants.

**Detailed review**

- Title: In my opinion, the title needs more clarity

**Abstract:**

- Well written

**Main script:**

Introduction: The introduction is clear, and the objectives are well-written. Some minor changes may be required. They are given below:

- The term *‘participants’* can be used in place of ‘subjects’ though out the manuscript
- In line 71, ‘numbers of patients’ should be changed to *‘number of patients’*
- In line nos. 80 and 82, ‘wearing a CI and a conventional hearing aid in opposite ears’ should be modified to ‘*wearing a CI in one ear and a conventional hearing aid in the opposite ear’*
- Justification for adding sound quality measurement is not given in the introduction
- Though introduction has clarity, justification for all the objectives need to be given, eg., for the last objective, ‘Evaluate if measuring time of loudness scaling can be reduced by using broadband instead of narrowband stimuli’
- The study involves adult listeners. But in the introduction, it is not clear whether the reviewed studies included adults or children.

Method:

- The method section explains the procedure well

Results and discussion:

- The results are explained in detail, and all the points are discussed adequately
- However, the result section is too lengthy

Figures:

- Could not view figure 3 in the manuscript
