# Supplementary material for: Extending the audiogram with loudness growth: The complementarity of electric and acoustic hearing in bimodal patients
Source: PLoS One. 2023 Apr 20;18(4):e0277161. doi: 10.1371/journal.pone.0277161 (PMC10118154; doi:10.1371/journal.pone.0277161)
Supplement: Attachment — Submitted filename: Response to reviewers.docx [file pone.0277161.s011.docx]

• In my opinion, the title needs more clarity

**We have changed the title to:**

**“Extending the audiogram with loudness growth: the complementarity of electric and acoustic hearing in bimodal patients”**

• The term *‘participants’* can be used in place of ‘subjects’ though out the manuscript

**As we believe the term ‘subjects’ fits better within the context of this article, we have not incorporated this comment.**

• In line 71, ‘numbers of patients’ should be changed to *‘number of patients’*

**Has been revised accordingly.**

•In line nos. 80 and 82, ‘wearing a CI and a conventional hearing aid in opposite ears’ should be modified to ‘*wearing a CI in one ear and a conventional hearing aid in the opposite ear’.*

**Has been revised accordingly.**

•Justification for adding sound quality measurement is not given in the introduction

**In line 91, we have now added:**

**‘Also, sound quality, which is often perceived as unnatural with CI alone [23], improved by adding a contralateral HA.’**

•Though introduction has clarity, justification for all the objectives need to be given, eg., for the last objective, ‘Evaluate if measuring time of loudness scaling can be reduced by using broadband instead of narrowband stimuli’.

**We have now rephrased line 141:**

**“Evaluate if measuring time of loudness scaling can be reduced by using broadband instead of narrowband stimuli, thereby reducing the amount of tests within the procedure.”**

**We believe the other study objectives are either self-evident or are sufficiently introduced within the introduction.**

• The study involves adult listeners. But in the introduction, it is not clear whether the reviewed studies included adults or children.

**Has been revised accordingly.**

• The result section is too lengthy

**We have shortened the results section by removing sections of the qualitative analysis and text within ‘Loudness and bimodal benefit’. If the reviewer believes the results section is still too lengthy, we could consider removing sections to the supplementary material.**

• Could not view figure 3 in the manuscript

**Figure 3 has been added in the original submission. Perhaps there were errors due to file size restrictions in the manuscript preparation. We have therefore also added Figure 3 below.**
